# Polar Layered Intermetallic LaCo_2_P_2_ as a Water Oxidation Electrocatalyst

**DOI:** 10.1021/acsami.1c19858

**Published:** 2022-03-15

**Authors:** Dallas K. Mann, Aida M. Díez, Junyuan Xu, Oleg I. Lebedev, Yury V. Kolen’ko, Michael Shatruk

**Affiliations:** † Department of Chemistry and Biochemistry, Florida State University, Tallahassee, Florida 32306, United States; ‡ International Iberian Nanotechnology Laboratory, Braga 4715-330, Portugal; § Laboratoire CRISMAT, UMR 6508, CNRS-Ensicaen, Caen 14050, France

**Keywords:** core−shell, electrocatalysis, intermetallic, oxygen evolution reaction, precatalyst, water oxidation

## Abstract

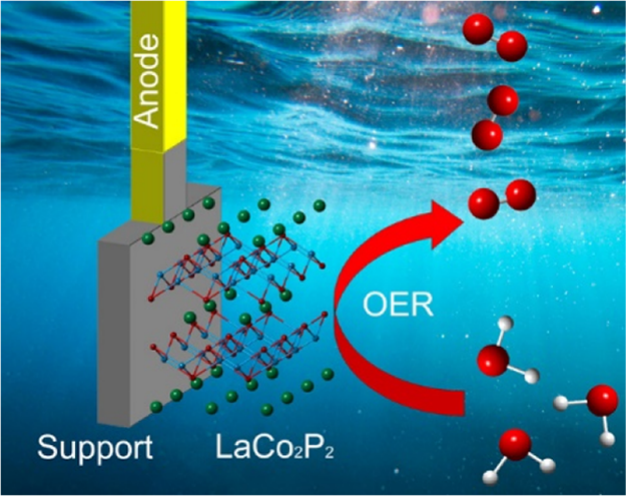

We investigate LaCo_2_P_2_ as an electrocatalytic material for oxygen evolution reaction (OER) under alkaline and acidic conditions. This layered intermetallic material was prepared *via* Sn-flux high-temperature annealing. The electrocatalytic ink, prepared with the ball-milled LaCo_2_P_2_ catalyst at the mass loading of 0.25 mg/cm^2^, shows OER activity at pH = 14, reaching current densities of 10, 50, and 100 mA/cm^2^ under the overpotential of 400, 440, and 460 mV, respectively. Remarkably, the electrocatalytic performance remains constant for at least 4 days. Transmission electron microscopy reveals the formation of a catalytically active CoO_*x*_ shell around the pre-catalyst LaCo_2_P_2_ core during the alkaline OER. The core serves as a robust support for the *in situ*-formed electrocatalytic system. Similar studies under pH = 0 reveal the rapid deterioration of LaCo_2_P_2_, with the formation of LaPO_4_ and amorphous cobalt oxide. This study shows the viability of layered intermetallics as stable OER electrocatalysts, although further developments are required to improve the electrocatalytic performance and increase the stability at lower pH values.

## Introduction

The growing worldwide need for clean energy technologies, aiming to replace rapidly depleting and environmentally harmful fossil fuels, has been driving extensive research efforts into the viability of a hydrogen fuel economy. The centerpiece of these efforts is the decomposition of water into the constituent elements.^1^ The overall water-splitting reaction can be divided into two half-reactions known as hydrogen evolution reaction (HER) and oxygen evolution reaction (OER). In practice, however, these electrochemical processes suffer from a large overpotential, defined as the difference between the experimental and thermodynamic values of the electrochemical potential required to drive the water electrolysis reaction. The overpotential is caused by substantial kinetic barriers associated with the two-electron HER and especially with the four-electron OER mechanisms.^2,3^ A broad range of electro- and photocatalysts has been investigated to address this problem.^4,5^ The state-of-the-art electrocatalysts, such as RuO_2_ and IrO_2_, have afforded a substantial decrease in the OER overpotential,^6^ but their use is not sustainable, given the scarcity and high cost of the platinum group metals.^7−9^ In recent years, extensive research efforts have been devoted to the discovery of sustainable electrocatalysts based on 3d metal hydroxides, oxyhydroxides, and oxides,^4,10−14^ chalcogenides,^15−17^ phosphides,^18−23^ and borides.^24,25^

The challenge in developing efficient electrocatalysts based on 3d metal compounds stems from the decomposition of such materials under conditions of electrocatalysis in acidic electrolytes. As a result, examples of such electrocatalysts that can operate under acidic OER conditions even for a few hours, without the loss of performance, are extremely rare. Among the materials mentioned above, transition-metal phosphides (TMPs) and transition-metal borides (TMBs) are well known to show high stability toward acids, in the absence of applied voltage, while OER activity in alkaline solutions has been demonstrated for several binaries, such as CoP, Ni_2_P, NiP_2_, FeB, Co_2_B, and Ni_2_B.^26−31^ Hence, TMPs and TMBs offer appealing alternatives to the more extensively studied OER electrocatalysts based on 3d metal hydroxides, oxyhydroxides, and oxides.^32^

**Figure 1:**
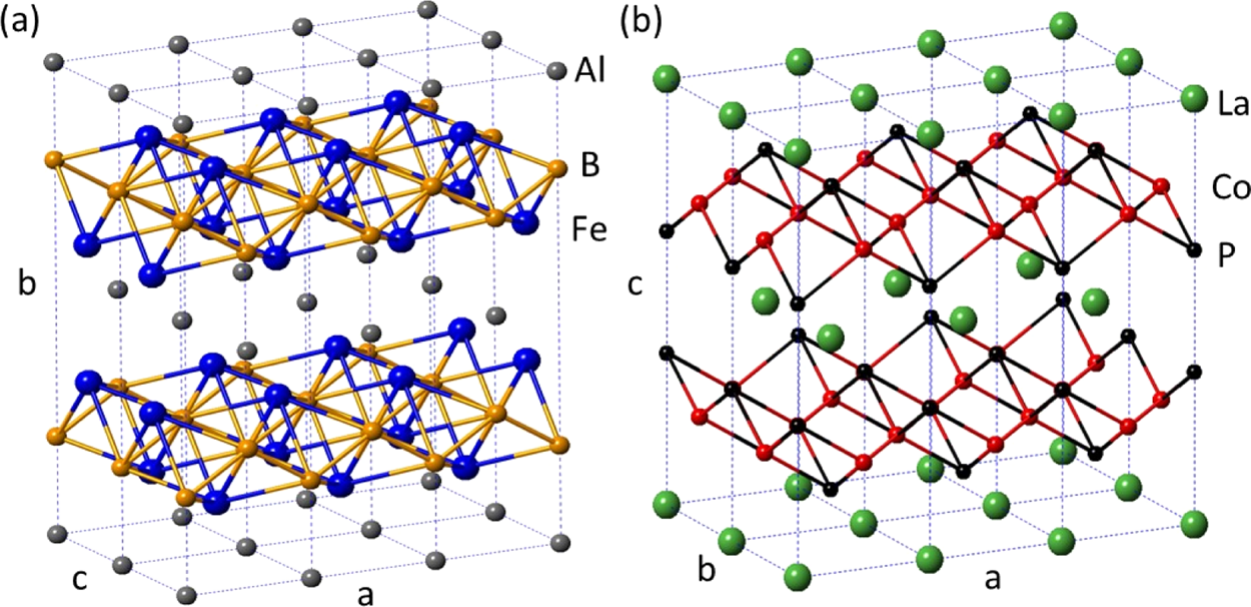
Side-by-side comparison of the crystal structures of AlFe_2_B_2_ (a) and LaCo_2_P_2_ (b).

Recently, we have reported a promising performance by AlFe_2_B_2_ in alkaline OER electrocatalysis.^29^ Our studies revealed that AlFe_2_B_2_ acts as a precatalyst by governing the formation of a thick and very stable shell of catalytically active Fe_3_O_4_ nanoparticles around the particles of AlFe_2_B_2_. Remarkably, this material far outperformed stand-alone Fe_3_O_4_ nanoparticles by showing a substantially higher long-term stability, faster reaction kinetics, and a lower overpotential in the electrocatalytic OER in a 1 M KOH electrolyte. Interestingly, AlFe_2_B_2_ also showed much better catalytic properties as compared to the Al-free FeB counterpart. We attributed this improved performance to the crystal structure of AlFe_2_B_2_, in which layers of Al atoms alternate with [Fe_2_B_2_] slabs (Figure 1a). The catalytically inactive Al layer provides additional structural stability and increased electrical conductivity within the structure, thus improving the electron-transfer kinetics and longevity of the catalytic system. At the onset of the OER, the surface layers of AlFe_2_B_2_ undergo reconstruction due to the partial etching of the Al layers by 1 M KOH and electro-oxidation of the [Fe_2_B_2_] slabs to the shell of Fe_3_O_4_ nanoparticles. Nevertheless, the remaining AlFe_2_B_2_ precatalyst core provides an excellent structural support and improved electron-transfer rate between the underlying electrode and the catalytically active oxide shell.

Despite its excellent performance in the alkaline electrocatalytic OER, AlFe_2_B_2_ is not stable under acidic conditions.^33−35^ In contrast, many ternary phosphides with related layered structures (Figure 1b) are known to exhibit high stability toward acids.^36^ Building on this knowledge, we have decided to explore the use of such structures in both acidic and alkaline water oxidation. Herein, we report a study of OER electrocatalysis on LaCo_2_P_2_, which was previously investigated by us and a few other research groups as an interesting ferromagnetic material with the ordering temperature of 132 K.^37−40^ We demonstrate that, despite its high general stability in acidic environment, this material quickly deteriorates once the voltage is applied to drive the OER at pH = 0. On the other hand, it acts as a stable OER precatalyst at pH = 14, showing only a slightly higher overpotential but similar electrochemical kinetics as compared to the reported performance of binary CoP. We also discuss the possible reasons for our observations and strategies to improve the stability and electrocatalytic performance of layered-structure TMPs.

## Materials and Methods

### Starting Materials

All manipulations during sample preparation were carried out in an Ar-filled dry box (content of O_2_ < 0.5 ppm). A lanthanum rod (≥99.9%), cobalt powder (99.9%), phosphorus powder (99.9%), tin powder (99.85%), and concentrated hydrochloric acid (36.5–38.0%) were obtained from VWR. The lanthanum rod was filed to powder immediately before the reaction. Cobalt powder was additionally purified by heating in a flow of H_2_ gas at 500 °C for 5 h. Nafion ionomer solution (5% in aliphatic alcohols and water) and platinum wire (99.9%) were acquired from Sigma-Aldrich, while the IrO_2_ (99.99%, #43396) reference material was purchased from Alfa Aesar. Ultrapure water (18.2 MΩ cm^–2^) was produced using a Milli-Q Advantage A10 system (Millipore). For electrolyte preparation, purified NaOH (98.5%) from Acros Organics and H_2_SO_4_ (95–98%) from Sigma-Aldrich were used.

### Synthesis

LaCo_2_P_2_ was synthesized according to the previously reported Sn-flux method.^37^ The starting materials were mixed in the La/Co/P/Sn = 1.6:2:2:20 ratio (the total mass = 2 g) and sealed in a fused silica tube of 10 mm inner diameter under vacuum (≈10^–4^ mbar). The mixture was annealed at 880 °C for 7 days and cooled to 600 °C at 10 °C/min, at which point the tube was quenched into ice water. The Sn-rich matrix was dissolved in dilute HCl (1:1 v/v) until the gas evolution ceased. The product was recovered by filtration, washed successively with water and ethanol, and dried.

Powder X-ray diffraction (PXRD) was carried out on a SmartLab diffractometer (Rigaku) equipped with a D/teX Ultra 250 1D detector and a Cu K_α_ radiation source (λ = 1.54187 Å). Each pattern was recorded in the 2θ range from 10 to 80° with a step of 0.03° and the total collection time of 1 h. The analysis of PXRD patterns was carried out with the SmartLab Studio II (Rigaku).^41^

### Electrocatalyst Preparation

The phase-pure sample of LaCo_2_P_2_ was ball-milled at 1725 rpm for 1 h in an 8000 M high-energy mixer/mill (SPEX), using a stainless steel ball-milling set. The milling process was performed under Ar atmosphere to minimize surface oxidation. The PXRD analysis of the ball-milled sample revealed no new impurity phases, although the diffraction peaks broadened, in accord with the decreased particle size and increased strain. The specific surface areas of the ball-milled LaCo_2_P_2_ and reference electrocatalyst were evaluated by measuring N_2_ physisorption using an Autosorb iQ2 analyzer (Quantachrome). For that purpose, ≈100 mg of a sample was placed in the sample holder tube and degassed at 120 °C for 2 h. Subsequently, the sample holder tube was placed into a liquid N_2_ bath for the analysis. The specific surface area of the materials was determined by the Brunauer–Emmett–Teller (BET) method.

The ball-milled sample of LaCo_2_P_2_ was converted to an electrocatalyst ink by dispersing 5 mg of the material in 50 μL of Nafion ionomer solution and 1.0 mL of anhydrous ethanol (Honeywell). The mixture was homogenized for 30 min in a bath sonicator USC-TH (VWR) and for 1 min with an ultrasonic probe Vibra-Cell 75185 (Thermo Fisher Scientific). Catalytic anodes were prepared by depositing the as-derived homogeneous ink on Ni foam (Heze Jiaotong, 110 pores per in., 0.3 mm thick) for alkaline OER or Ti felt (Bekaert Fibre Technologies, 20 μm fiber diameter, 1 mm thick) for acidic OER. Before the ink deposition, both the Ni foam and the Ti felt were cleaned by sequential 30 min ultrasonication in acetone, ethanol, and Milli-Q water. Prior to ultrasonic cleaning, the Ti felt was additionally purified by heating it under Ar atmosphere to 400 °C at 3 °C/min and maintaining it at this temperature for 2 h. In each case, 640 μL of the ink was loaded in 20 μL batches on the surface of the Ni foam or the Ti felt current collectors, letting ethanol evaporate between the batches. The exposed surface area of the anode was fixed to be 1 cm^2^, and the total mass of the ball-milled LaCo_2_P_2_ catalyst or the reference IrO_2_ catalyst loaded on the anode was varied from 0.25 to 3 mg/cm^2^. Finally, the obtained anode was air-dried.

### Electrocatalytic Measurements

Electrochemical studies were conducted at room temperature using an Autolab PGSTAT302N potentiostat (Metrohm). The OER performance of the electrocatalysts was evaluated under moderate Ar bubbling (≈1 bubble/s) while stirring at 150 rpm in a three-electrode system filled with a purified 1.0 M NaOH (alkaline OER) or 0.5 M H_2_SO_4_ (acidic OER) electrolyte. The catalytic anode, a calibrated saturated calomel electrode (SCE), and a Pt wire served as the working, reference, and counter electrodes, respectively. Unless stated otherwise, all potentials reported in this work were converted to a reversible hydrogen electrode (RHE) reference scale according to the following equation: *E*_RHE_ = *E*_SCE_ + 0.059pH + 0.241. An *iR* correction of 85% was applied in the polarization experiments to compensate for the voltage drop between the reference and working electrodes, which was evaluated by a single-point high-frequency impedance measurement. OER anodic polarization curves were recorded using cyclic voltammetry (CV) with a scan rate of 5 mV/s. In the case of electrocatalyst activation, the scan rate was augmented to 50 mV/s. The catalytic stability of the anodes was evaluated as a function of time by means of chronopotentiometry at a constant current density of 10 mA/cm^2^.

Electrochemical impedance spectroscopy (EIS) was carried out on stable electrocatalytic systems at an overpotential of 0.42 V in the frequency range from 105 to 0.01 Hz with a 10 mV sinusoidal perturbation. The EIS measurements and the interpretation of results were conducted in accordance with an aqueous electrochemical assembly, the so-called supported system.^42^

### Chemical Analysis

Inductively coupled plasma-optical emission spectroscopy (ICP-OES) was carried out using an ICPE-9000 spectrometer (Shimadzu). Each sample was measured three times to ensure the reproducibility of results.

### Transmission Electron Microscopy

Transmission electron microscopy (TEM), high-resolution TEM (HRTEM), high-angle annular dark-field scanning TEM (HAADF-STEM), selected area electron diffraction (SAED), and energy-dispersive X-ray spectroscopy in STEM mode (STEM–EDX) were performed using a JEM-ARM200F microscope (JEOL) equipped with a cold field-emission gun, a probe, an image aberration correction, a CENTURIO EDX detector, and a GIF Quantum filter. TEM samples were prepared by crushing a sample in an agate mortar in ethanol and depositing the obtained suspension on a copper carbon holey grid.

## Results and Discussion

### Electrocatalyst Preparation

**Figure 2:**
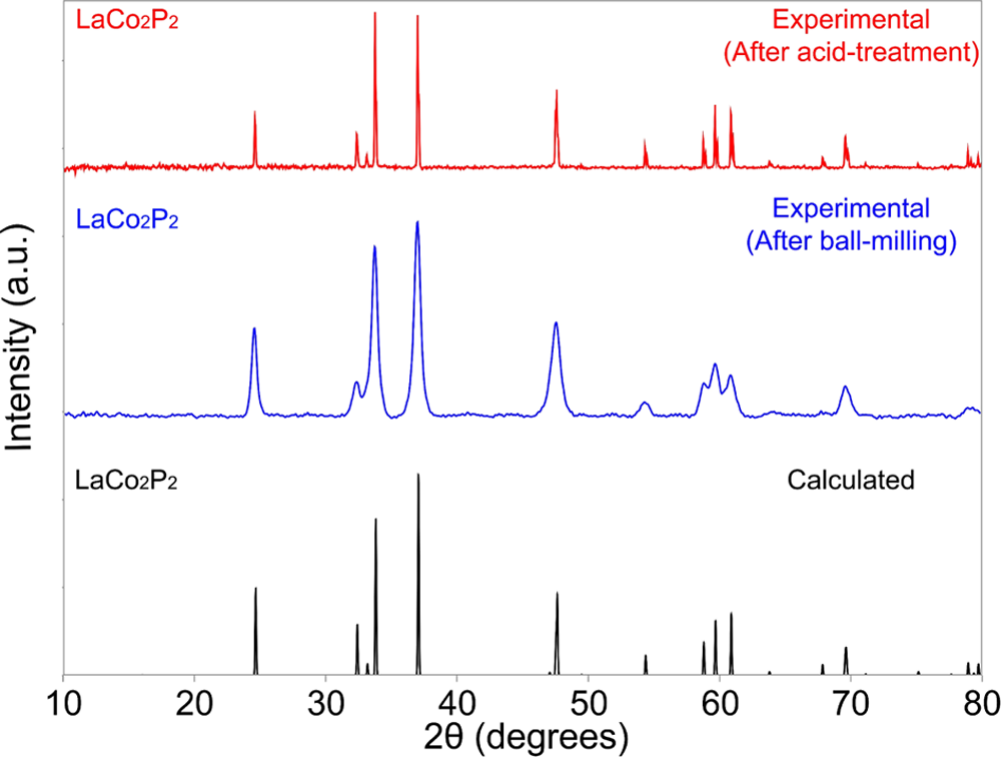
PXRD patterns for the bulk (red) and ball-milled (blue) samples of LaCo_2_P_2_. The calculated pattern (black) for LaCo_2_P_2_ is provided for comparison.

Bulk LaCo_2_P_2_ was prepared by annealing the constituent elements in Sn flux. After the Sn-rich matrix had been dissolved in dilute HCl, a phase-pure material was obtained, as evidenced by PXRD (Figure 2). The material was ball-milled for 1 h to increase the surface area for improved catalytic performance. According to the BET method, the surface area of the ball-milled sample was 9.9 cm^2^/g. PXRD of the ball-milled material revealed that the sample remained phase-pure, but the diffraction peaks broadened due to the well-known combined effects of the decreased particle size and the strain introduced by ball-milling. The crystallite size was estimated as *D* = *K*λ/[(*w*_s_ – *w*_i_)·cos(θ)],^43^ where *K* is a shape factor (set to 0.9), λ is the X-ray wavelength, θ is the diffraction angle of the observed peak, and *w*_s_ and *w*_i_ are the full widths at half-maximum for the sample and an instrumental standard (highly crystalline Si powder), respectively. Using this equation, the lower bound for the crystallite size was estimated at ≈25 nm. This value does not represent the actual average crystallite size because the Scherrer equation does not account for the strain induced by the ball-milling process, which will necessarily increase the *w*_s_ value.

**Figure 3:**
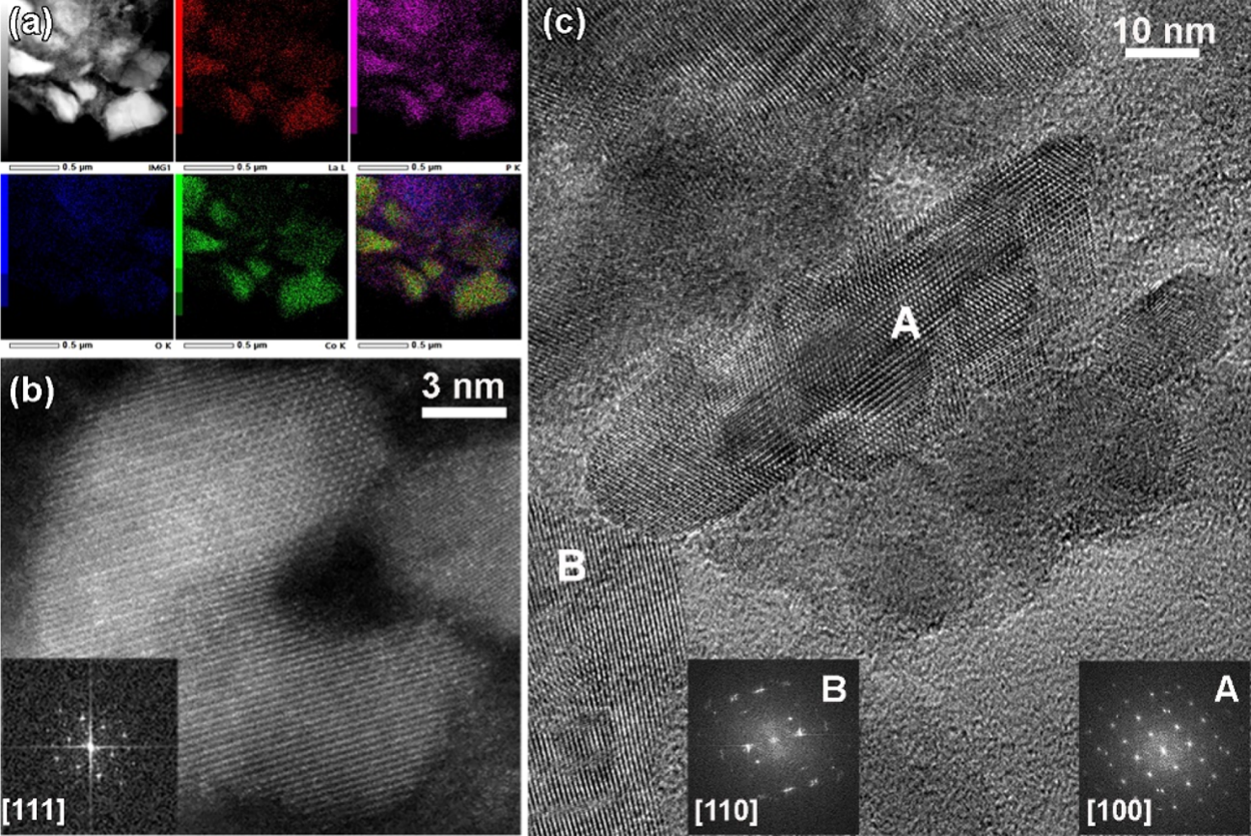
TEM analysis of LaCo_2_P_2_ particles after ball-milling: (a) low-magnification overview HAADF-STEM image and simultaneous STEM–EDX elemental mappings acquired at the L-line of La (red) and K-lines of P (purple), O (blue), and Co (green), and their mixture; (b) high-resolution HAADF-STEM image of a single LaCo_2_P_2_ particle viewed along the [111] zone axis (the corresponding FT pattern is shown in the inset); (c) bright-field HRTEM image of the LaCo_2_P_2_ sample, together with the insets showing the FT patterns taken from [100] (A) and [110] (B) oriented particles.

The TEM characterization prior to electrochemical testing revealed that the sample of ball-milled LaCo_2_P_2_, in general, showed good crystallinity and contained a mixture of nanosized (≈10 nm) and submicron particles. The STEM–EDX elemental mappings (Figure 3a) indicate homogeneous chemical distribution of all constituent elements (La, Co, and P), as well as a negligible amount of oxygen that can be attributed to minor surface oxidation. High-resolution HAADF-STEM (Figure 3b) and TEM (Figure 3c) images, along with the corresponding Fourier transform (FT) patterns, confirm the tetragonal structure of LaCo_2_P_2_ (space group *I*4/*mmm*, *a* = 3.8145 Å, *c* = 11.041 Å, ICSD-624010).

### Electrochemical Testing

**Figure 4:**
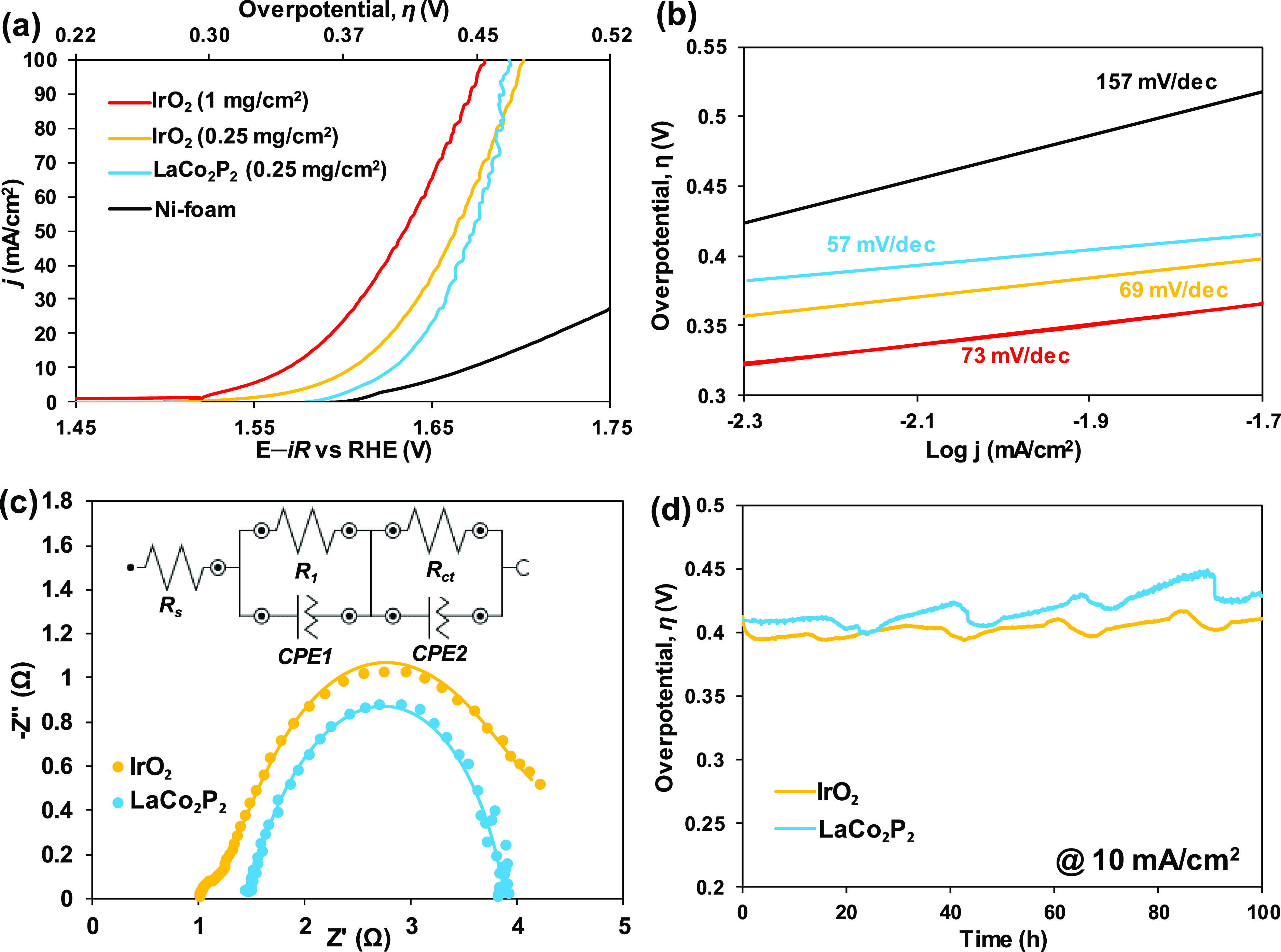
(a) Alkaline OER anodic polarization curves for Ni foam (after 100 activation cycles), Ni foam-supported ball-milled LaCo_2_P_2_ at 0.25 mg/cm^2^ loading (after 100 activation cycles) and Ni foam-supported reference IrO_2_ catalyst at 0.25 and 1 mg/cm^2^ loadings. (b) Respective Tafel plots. (c) Comparison of Nyquist plots for Ni foam-supported LaCo_2_P_2_ and Ni foam-supported reference IrO_2_ at the applied overpotential η = 420 mV. The inset shows an equivalent electrical circuit model used to fit the Nyquist plots. (d) Chronopotentiometric stability tests under alkaline OER for Ni foam-supported LaCo_2_P_2_ and Ni foam-supported reference IrO_2_ at the same loading of 0.25 mg/cm^2^.

The electrochemical OER testing in a purified 1 M NaOH electrolyte revealed that LaCo_2_P_2_ requires initial activation for *ca.* 100 CV cycles to achieve steady-state conditions, after which the material exhibits constant OER performance. Experimentally, it was found that LaCo_2_P_2_ with a loading of 0.25 mg/cm^2^ was the most OER-active (Figure S1), reaching anodic current densities of 10, 50, and 100 mA/cm^2^ at overpotentials of *ca.* 400 mV (η_10_), 440 mV (η_50_), and 460 mV (η_100_), respectively (the blue curve in Figure 4a). A reference state-of-the-art IrO_2_ catalyst (Figure S2) showed a higher performance at an industrially relevant loading of 1 mg/cm^2^ (the red curve in Figure 4a), but at the same loading as that of LaCo_2_P_2_, that is, at 0.25 mg/cm^2^, the overpotentials achieved with IrO_2_ were quite similar (the yellow curve in Figure 4a). We would like to emphasize that at the same loading of 0.25 mg/cm^2^, the BET surface area of the IrO_2_ catalyst (25.5 cm^2^/g) was substantially larger than that of the LaCo_2_P_2_ material (9.92 cm^2^/g), while the cost of the IrO_2_ catalyst at such loading would be prohibitively high. Nevertheless, it might still be possible to achieve lower overpotentials with the LaCo_2_P_2_-based catalyst, if the particle size can be further reduced to increase the electrocatalytically active surface area. Such an effect could be achieved, for example, by a solvent-assisted ball-milling process.

Given these promising results, the kinetic behavior of LaCo_2_P_2_ was investigated using a combination of Tafel and Nyquist plots. The Tafel plot relates the rate of the electrochemical reaction to the overpotential as η = *b* log(*j*/*j*_0_), where *j* is the current density, *j*_0_ is the exchange current density (*i.e.*, the current density at zero overpotential), and *b* is the Tafel slope, which represents the decade change in the reaction rate as a function of applied voltage.^44^ Analysis of the electrocatalytic data according to this equation led to *b* = 57 mV dec^–1^ for LaCo_2_P_2_ and *b* = 69 mV dec^–1^ for IrO_2_ at the same 0.25 mg/cm^2^ loading (Figure 4b). Subsequently, EIS data were collected at low frequencies (Figure 4c), and the obtained semicircles were fit with an equivalent circuit model (Figure 4c, inset) consisting of a resistor (*R*_s_) in series with two parallel combinations of a resistor (*R*_1_, *R*_ct_) and a constant phase element (CPE1, CPE2).^45^*R*_s_ represents the Ohmic resistance from the electrolyte and all contacts. The time constant *R*_1_–CPE1 accounts for the interfacial resistance from the electron transport between the LaCo_2_P_2_ material and supporting Ni foam. *R*_ct_–CPE2 is the charge-transfer resistance (*R*_ct_) at the interface between the catalyst and the electrolyte, and smaller *R*_ct_ values typically reflect faster charge-transfer kinetics. The derived parameters, listed in Table 1, reveal that the LaCo_2_P_2_ anode shows a significantly smaller *R*_ct_ value (1.470 Ω) as compared to that of IrO_2_ (2.820 Ω), indicating that the LaCo_2_P_2_ catalyst offers favorable charge-transfer kinetics, competitive with that of IrO_2_.

**Table 1:** Impedance Parameters Calculated by Fitting the Nyquist Plots (Figure 4c)

circuit element	LaCo_2_P_2_	IrO_2_
equivalent series resistance (*R*_s_)	1.460 Ω	0.985 Ω
electron-/charge-transport resistance (*R*_1_)	0.961 Ω	1.860 Ω
charge-transfer resistance (*R*_ct_)	1.470 Ω	2.820 Ω

Lastly, we performed chronopotentiometry to study the stability of LaCo_2_P_2_ and reference IrO_2_ with the same mass loading of 0.25 mg/cm^2^ under the alkaline OER conditions (pH = 14). Over 100 h, LaCo_2_P_2_ compares favorably to IrO_2_ and, more importantly, demonstrates an essentially constant performance for more than 4 days, driving the current density of 10 mA/cm^2^ with η ≈ 400 mV in the 1 M NaOH electrolyte solution (Figure 4d). Notably, the ICP-OES chemical analysis of the electrolyte after the stability testing showed the presence of only 0.077 ppm of Pt admixture, which, most likely, stems from leaching of the counter electrode.^46^ Importantly, no traces of La, Co, or Ni were identified by the chemical analysis of the alkaline electrolyte, suggesting excellent stability of the LaCo_2_P_2_ catalyst, in particular, and the LaCo_2_P_2_/Ni foam anode assembly, in general.

**Figure 5:**
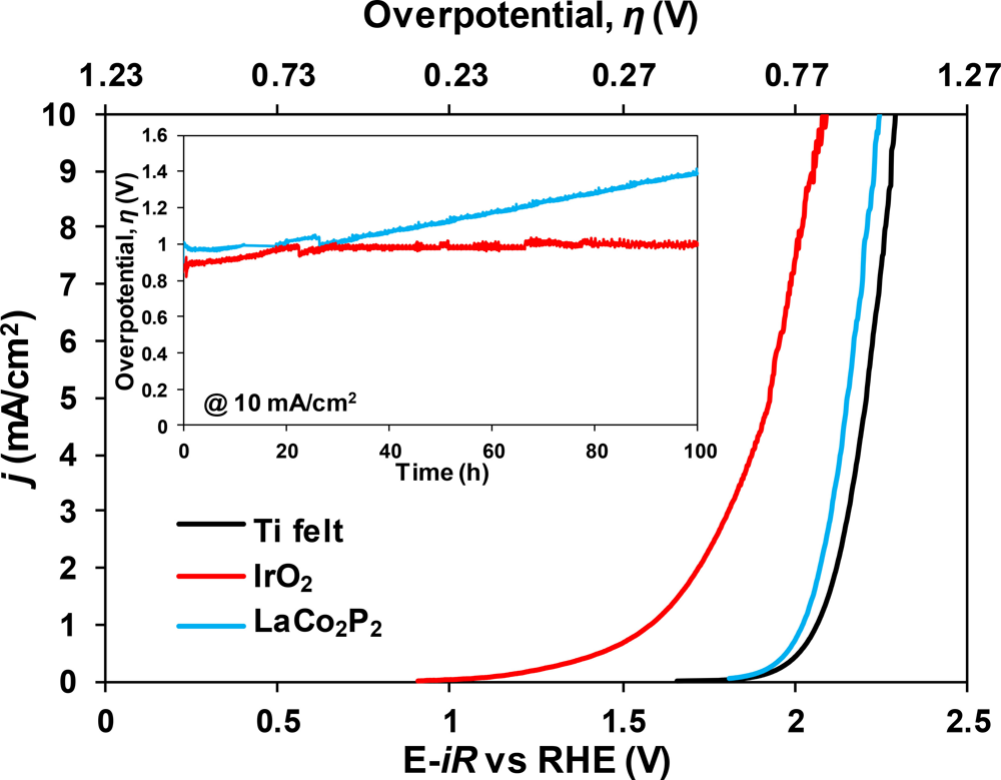
Acidic OER anodic polarization curves and the continuous chronopotentiometric profiles (shown as an inset) for the Ti felt, as well as Ti felt-supported ball-milled LaCo_2_P_2_, and Ti felt-supported reference IrO_2_ materials, both at 3 mg/cm^2^ loading.

After the studies of LaCo_2_P_2_ under alkaline OER, the material was tested under acidic OER (pH = 0) to determine its possible bifunctionality. The electrochemical station and the sample were prepared in a manner identical to that described for the alkaline OER, with the catalyst ink deposited on a Ti felt and 0.5 M H_2_SO_4_ used as the electrolyte. Under such conditions, the LaCo_2_P_2_ catalyst showed a much higher overpotential, η = 1.01 V, at 10 mA/cm^2^ (Figure 5). Chronopotentiometric testing (Figure 5, inset) for driving current density of 10 mA/cm^2^ showed that LaCo_2_P_2_ is not stable, causing a gradual increase in the overpotential with time. Thus, this material is impractical as an OER electrocatalyst under acidic conditions.

### Post-electrochemical Testing

#### PXRD

**Figure 6:**
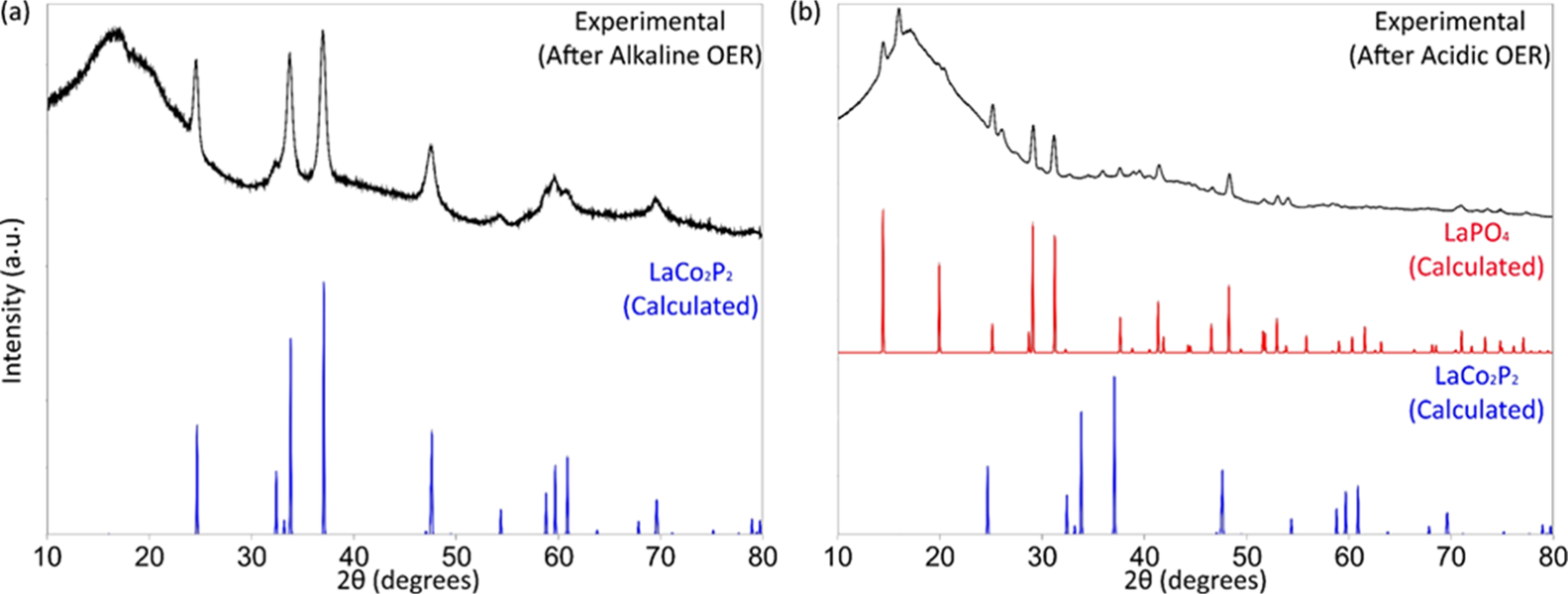
PXRD patterns of LaCo_2_P_2_ after 100 h of chronopotentiometric testing at 10 mA/cm^2^ in 1 M NaOH (a) and 0.5 M H_2_SO_4_ (b). Broad amorphous peaks present in both samples are due to the Nafion ionomer. The calculated patterns for LaCo_2_P_2_ and LaPO_4_ are shown as references.

To monitor changes to the LaCo_2_P_2_ phase under the OER conditions, the samples obtained after electrochemical testing in the alkaline and acidic electrolytes were harvested from the Ni foam and Ti felt, respectively, and subjected to PXRD analysis. The PXRD patterns obtained for the sample used in alkaline OER revealed that LaCo_2_P_2_ remains stable under the harsh alkaline conditions (Figure 6a). The large unresolved amorphous peaks that appear in the low-angle region are due to a Nafion ionomer admixture in the postcatalytic sample. The lower bound for the crystallite size, estimated from the Scherrer equation, decreased from 25 nm before the catalysis to 19 nm after the catalysis. This change is in agreement with the OER-induced *in situ* surface reconstruction that converts the outer layers of the LaCo_2_P_2_ particles to a shell of a catalytically active oxide–(oxy)hydroxide phase,^29,32^ as suggested by the observations made in the TEM studies discussed below.

In sharp contrast to the alkaline OER testing, the sample obtained after the acidic OER testing contained predominantly LaPO_4_ and minor impurities that could not be assigned due to the large amorphous peak observed in the low-angle region due to the Nafion ionomer (Figure 6b). As seen later from the EDX mapping results, the other impurity is Co-based due to the high content of Co found in this sample.

#### Electron Microscopy

**Figure 7:**
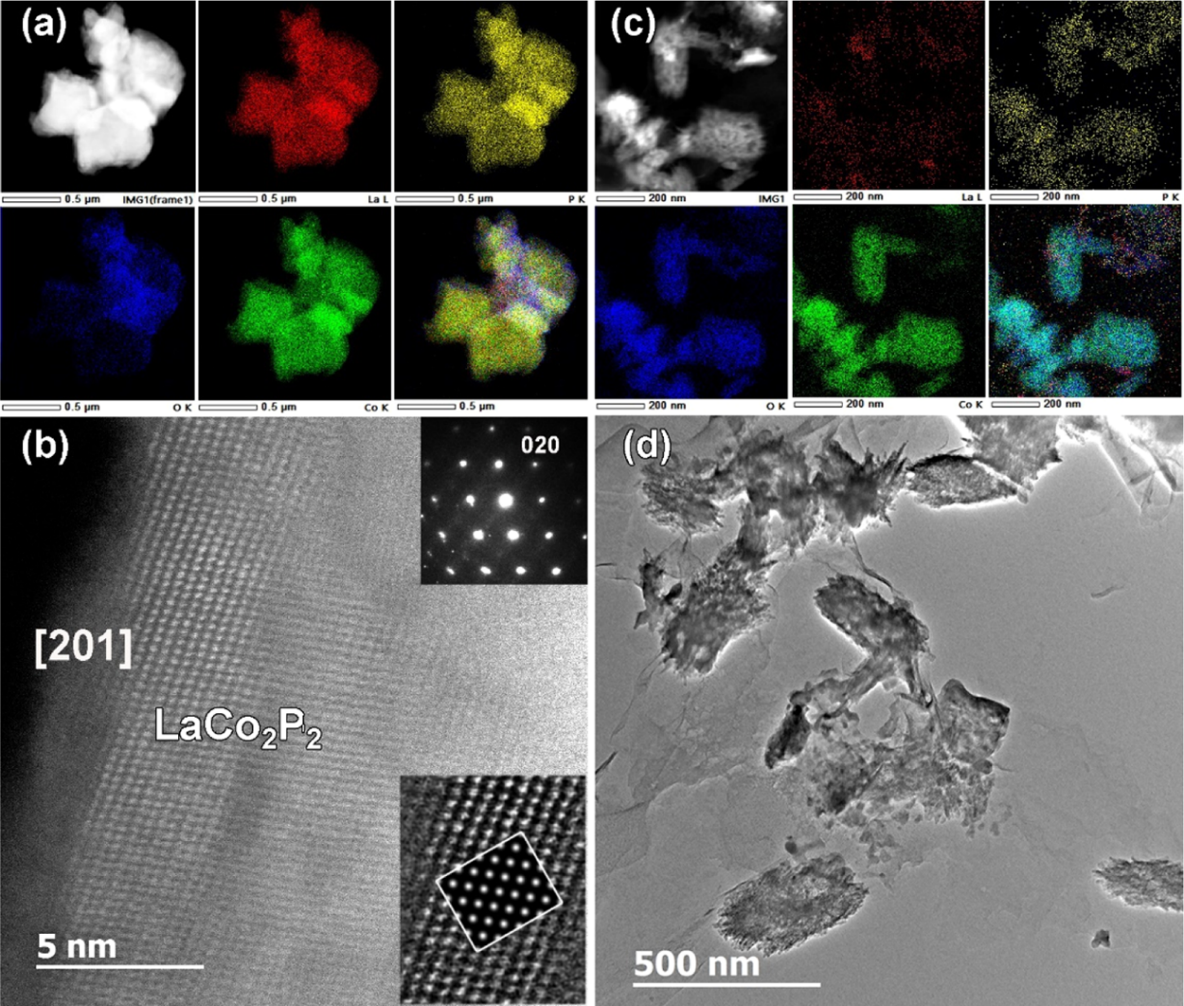
HAADF-STEM images together with simultaneously collected STEM–EDX elemental mappings of La, P, O, Co, and their mixture for LaCo_2_P_2_ particles after OER electrocatalysis in alkaline (a) and acidic (c) electrolytes. (b) High-resolution [201] HAADF-STEM image with the corresponding SAED (upper corner inset) and the magnified HAADF-STEM image with an overlaid simulated image (bottom corner inset) for LaCo_2_P_2_ particles after alkaline OER electrocatalysis. (d) Low-magnification TEM overview image for the LaPO_4_ needle-like particles formed after acidic OER electrocatalysis over LaCo_2_P_2_.

The samples obtained after the alkaline and acidic OER testing were subjected to TEM imaging and STEM–EDX elemental mapping. In comparison to the sample prior to testing (Figure 3), an extreme increase in the oxygen content was observed after 100 h of stability testing under alkaline conditions (Figure 7a). HAADF-STEM and SAED were employed to determine the nature of the oxygen-containing phase formed on the surface of the LaCo_2_P_2_ particles. These particles showed well-defined lattice planes (Figure 7b) with a number of defects, while the respective SAED (Figure 7b, inset) could be indexed with the lattice parameters corresponding to the tetragonal *I*4/*mmm* structure of LaCo_2_P_2_ (*a* = 3.814 Å, *c* = 11.041 Å, ICSD-624010). The HAADF-STEM image simulation based on this structure showed a good agreement with the experimental one (Figure 7b, inset). Interestingly, the edge of the LaCo_2_P_2_ particle is decorated by an amorphous layer of several nanometers thickness, and according to STEM–EDX elemental mapping, it can be attributed to the CoO_*x*_ phase. The high-resolution HAADF-STEM image (Figure 7b) clearly reveals a transition from the region of the CoO_*x*_ shell to the layered structure of the LaCo_2_P_2_ core, indicating the *in situ* surface reconstruction caused by alkaline OER electrocatalysis.

Under acidic OER conditions, the LaCo_2_P_2_ catalyst underwent a complete change in its morphology, as compared to the material before electrocatalysis. The particle shape changed from plate-like (Figure 3a) to needle-like (Figure 7c). This observation is in agreement with the formation of LaPO_4_ as the major phase observed by PXRD (Figure 6b). Indeed, LaPO_4_ was shown to form rodlike particles^47,48^ that exhibit an increasing aspect ratio with decreasing pH.^49^ The STEM–EDX elemental mapping of the postcatalytic sample showed an increase in the O content and a decrease in the La and P contents, while the content of Co remained relatively high (Figure 7c). These observations support the PXRD findings, which showed that LaCo_2_P_2_ decomposes during the acidic OER electrocatalysis to form LaPO_4_, and also suggest that the crystalline phosphate particles are surrounded by an amorphous cobalt oxide phase. This phase was unstable under the electron beam during TEM measurements even at low voltage (80 kV), which impeded the high-resolution imaging to identify the exact nature of this oxidic Co phase.

## Concluding Remarks

Based on the results presented above, we rationalize that LaCo_2_P_2_ acts as pre-catalyst in the alkaline water oxidation. Under the applied voltage at pH = 14, its surface undergoes *in situ* reconstruction, most likely, according to the following equation
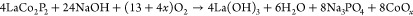


Although this equation is a much-simplified version of what actually occurs on the surface, it allows us to highlight two major points. First, during approximately the first 100 CV cycles of required activation, as established experimentally, the surface of the LaCo_2_P_2_ particles is converted to the oxidic CoO_*x*_ shell. The formation of the La(OH)_3_ phase is postulated according to the Pourbaix diagram for La at the specific applied voltage and pH = 14.^50^ Second, the surface is reconstructed to the catalytically active amorphous CoO_*x*_ shell (or perhaps the CoOOH shell^51^), as evidenced by the electron microscopy analysis and the experimentally observed decrease in the overpotential over the first 100 cycles of the electrocatalysis. While CoO_*x*_ serves as an active OER electrocatalyst, the underlying LaCo_2_P_2_ phase provides a robust support, allowing the catalyst to maintain stability for at least 4 days of electrocatalysis. In contrast, under acidic conditions, LaCo_2_P_2_ rapidly decomposes to LaPO_4_ and amorphous cobalt oxide or (oxy)hydroxide, resulting in a rapid increase in the overpotential.

To determine the effectiveness of LaCo_2_P_2_ as an alkaline OER electrocatalyst, we compared its performance to that of other Co-based OER electrocatalysts studied under alkaline conditions (Table 2). The overpotential at 10 mA/cm^2^ (η_10_ = 400 mV) observed for the CoO_*x*_/LaCo_2_P_2_ electrocatalytic system in the present work is slightly higher than the values reported for other Co-based electrocatalysts, which typically show η_10_ above 300 mV. On the other hand, the Tafel slope of 57 mV, measured for CoO_*x*_/LaCo_2_P_2_ in this work, is comparable to the values observed for the other Co-based systems at similar catalyst loading. Xing *et al.* added polythiophene to increase the electrical conductivity of the catalyst to achieve an overpotential of 338 mV at 10 mA/cm^2^ and a low Tafel slope of 52 mV/dec.^52^ Nevertheless, the stability of such a system is substantially lower than that of LaCo_2_P_2_, as the performance decreased by 5% after 15 h, whereas our LaCo_2_P_2_ catalyst maintained constant performance for 100 h (Figure 4a, inset). Li *et al.* achieved good electrocatalytic results by applying an external magnetic field during the synthesis of the catalyst (Ni–S–CoFe_2_O_4_), which they attributed to the formation of a larger concentration of catalytically active sites and an effective electrochemical surface area on the active site surface.^53^

**Table 2:** Overpotential and Performance Parameters Reported for Cobalt Oxides and Phosphides Used in Alkaline OER Electrocatalysis

catalyst	electrolyte	catalyst loading (mg/cm^2^)	η_10_ (mV)	*b* (mV/dec)	references
**LaCo**_**2**_**P**_**2**_	**1 MNaOH**	**0.25**	**400**	**57**	**this work**
IrO_2_	1 M NaOH	1.0	340	73	this work
IrO_2_	1 M NaOH	0.25	380	69	this work
co-polythiophene	1 M KOH	1.4	338	52	(52)
Ni–S–CoFe_2_O_4_	1 M KOH	n/a	228	72	(53)
La_0.9_CoO_3−δ_	0.1 M KOH	0.24	380	83	(54)
Co_3_O_4_ (nanoparticles)	0.1 M KOH	n/a	310	53	(55)
CoP (film)	1 M KOH	2.5	345	47	(56)
CoP (nanoframes)	1 M KOH	0.27	323	50	(57)
CoP (N-doped carbon)	1 M KOH	0.27	354	60	(57)
CoP (graphitic carbon)	1 M KOH	n/a	345	56	(58)

Overall, the performance of LaCo_2_P_2_ in OER electrocatalysis is comparable to the previously reported performance by CoP, as well as to the performance of the IrO_2_ benchmark. The presence of the additional La layer does not have a substantial effect on the electrochemical activity, which is in contrast to our recent finding for AlFe_2_B_2_,^29^ where the extra layer of Al appeared to improve the charge-transfer kinetics in comparison to the performance of binary iron borides. The lack of improved kinetics may be attributed to the higher polarity of the LaCo_2_P_2_ structure that contains alternating electropositive La layers and electronegative [Co_2_P_2_] layers. Another detrimental factor might be the presence of insoluble La(OH)_3_ in the amorphous surface layer. Lowering the polarity of the intermetallic structure and using an electropositive element that forms a more soluble hydroxide might alleviate these problems. In this vein, ACo_2_P_2_ structures that contain alkaline-earth metals (A) can serve as promising targets for future studies. These efforts are currently underway in our laboratories, and the results or such studies will be reported in due course.

## Supporting Information Available

Alkaline OER activity of Ni foam-supported ball-milled LaCo_2_P_2_ and reference IrO_2_ catalyst as a function of the material mass loading (PDF)
