# Supplementary material for: Polar Layered Intermetallic LaCo2P2 as a Water Oxidation Electrocatalyst
Source: ACS Appl Mater Interfaces. 2022 Mar 15;14(12):14120–8. doi: 10.1021/acsami.1c19858 (PMC9455929; doi:10.1021/acsami.1c19858)
Supplement: Supplementary file 1 — am1c19858_si_001.pdf [file am1c19858_si_001.pdf]

Supporting Information

Polar Layered Intermetallic $LaCo_2P_2$ as Water Oxidation Electrocatalyst

Dallas K. Mann,[a] Aida M. Díez,[b] Xu Junyuan,[b] Oleg I. Lebedev,[c] Yury V. Kolen'ko,[b,*] Michael Shatruk[a,*]

[a] *Department of Chemistry and Biochemistry, Florida State University, Tallahassee, FL 32306, United States*
[b] *International Iberian Nanotechnology Laboratory, Braga 4715-330, Portugal*
[c] *Laboratoire CRISMAT, UMR 6508, CNRS-Ensicaen, Caen 14050, France*

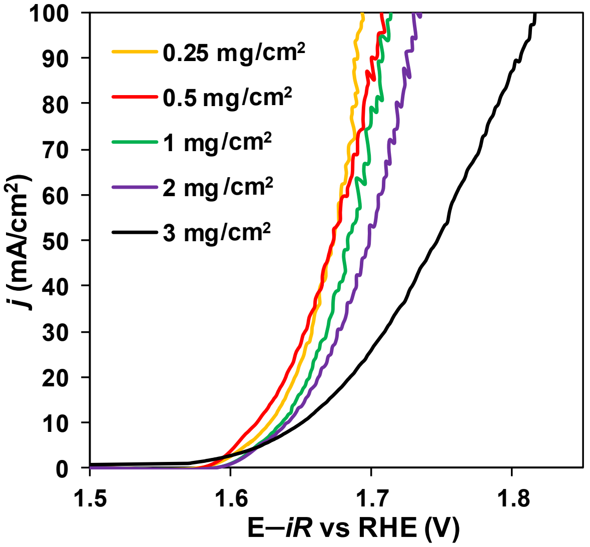

Figure S1. Alkaline OER activity of Ni-foam-supported ball-milled $LaCo_2P_2$ as a function of the material mass loading. Anodic polarization curves are recorded after 100 CV cycles of activation.

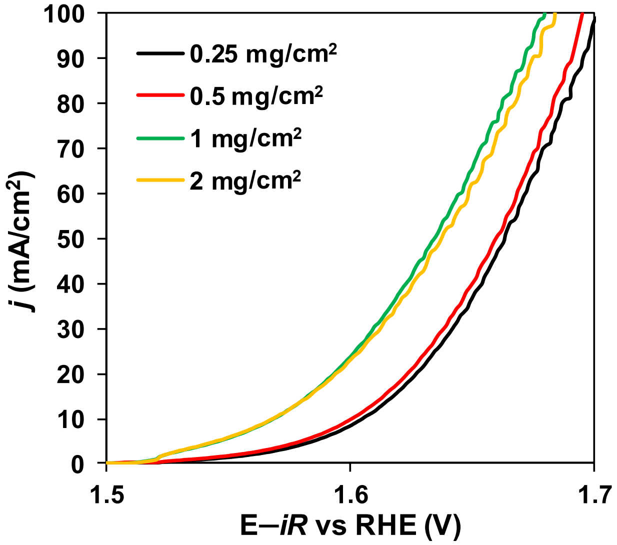

Figure S2. Alkaline OER activity of the Ni-foam-supported reference IrO_2 catalyst as a function of the material mass loading.
